# Editorial: New challenges and future perspectives in Alzheimer's disease and related dementias

**DOI:** 10.3389/fnagi.2023.1345560

**Published:** 2023-12-12

**Authors:** Gong-Ping Liu, Shuo Wang

**Affiliations:** ^1^Co-Innovation Center of Neuroregeneration, Nantong University, Nantong, China; ^2^Key Laboratory of Ministry of Education of China and Hubei Province for Neurological Disorders, Department of Pathophysiology, School of Basic Medicine, Tongji Medical College, Huazhong University of Science and Technology, Wuhan, China; ^3^Huffington Center on Aging, Baylor College of Medicine, Houston, TX, United States

**Keywords:** editorial, Alzheimer's disease, related dementias, new challenges, future perspectives

Dementia affects more than 55 million people worldwide, currently making it the seventh -leading cause of death globally (Alzheimer's & Dementia: Global Resources). Alzheimer's disease (AD) is the most common cause. Over the last decade, there has been growing interest and important developments in Alzheimer's disease (AD) and related dementias researches. These advances have been fostered by improvements in the research techniques that enable us to gather insights from different approaches. For examples, biomarkers including new brain-imaging techniques such as Positron Emission Tomography (PET) scans, and new methods to analyze cerebrospinal fluid have helped researchers to detect early changes in brain function in people with Alzheimer's. However, there are still numerous mysteries that remain unsolved, what will be a safe and effective treatment for Alzheimer's disease related dementia? What is the relationship between the plaques and the tangles found in the brain? What does inflammation have to do with Alzheimer's disease and related dementia?

In this Research Topic, researchers are encouraged to sum up the key challenges and research achievements in the research domain of AD and related dementias these years, and future research directions and prospects in the next few years are also welcomed to devote. Through a long process of strategizing, organizing, soliciting and publishing, it is expected to have 28 manuscripts, and 20 manuscripts were submitted, finally, only four manuscripts were published, which have been fully viewed more than 14 K times, over 2,889 downloads and cited by six times until now.

In this issues of frontiers, Manippa et al. summarized the research advances in gamma entrainment using sensory stimulation (GENUS) and the evidences of GENUS for AD treatment in AD transgenic mice and patients. Though the application has just started in human, sensory stimulation effectiveness have shown to improve cognition, with reducing Abeta burden and phosphorylated tau levels in animal models, and displaying a trend of down regulation of immunologic factors in AD patients' brains (Intlekofer and Cotman, 2013; Yi et al., 2015). They also emphasized that, further research is needed to develop GENUS as a non-drug intervention for the disease.

Several studies have revealed the relationship between osteoporosis and AD (Tolppanen et al., 2013; Dengler-Crish and Elefteriou, 2019), However, Hu et al. used a two-sample Mendelian randomization to test the causal link in either direction between osteoporosis and AD, and they found no genetic causality between them. This study provides us a new but contradictory perspective on this relationship, letting us to pursue more studies and discussions for the conclusion.

As the two most common dementias of neurodegeneration, AD and Lewy body disease (LBD) can occur as a complication (AD + LBD); it is known that dementia pedigree and demographic variables could cause diagnostic uncertainty, however, the magnitude of this uncertainty is not clear. Wei et al. compared clinical diagnosis with postmortem examination-confirmed pathological to evaluate the quality of the clinical subtype diagnosis, using the recorded data of 1,920 patients come from the National Alzheimer's Coordinating Center from 2005 to 2019. They found that clinical diagnosis of AD + LBD had poor sensitivities. Over 61% of participants with autopsy-confirmed AD + LBD were diagnosed clinically as AD. Among participants diagnosed as AD in the clinic, over 32% had concurrent LBD neuropathology at autopsy. 32 to 54% revealed concurrent autopsy-confirmed AD pathology among participants diagnosed as LBD. They concluded that clinical diagnosis of AD, LBD, and AD + LBD are inaccurate and suffer from significant disparities on race and sex. They provided important implications for clinical management and applicability of potential therapies for AD.

The earliest clinical symptom of the preclinical phase of AD has recognized as subjective cognitive decline (SCD) with a positive amyloid burden, Sun et al. found that individuals with SCD may have subclinical episode memory defects, which related to the Abeta burden in the whole brain. They also revealed that, compared with AD individuals and the healthy older adults, the increase white matter microstructural integrity in the right cingulum of SCD individuals might compensate for short-term episode memory. These data supply an interesting interventional target for further studies.

The papers in this Research Topic helped us to get a broader and deeper understanding of diagnosis and treatment of AD and other dementias, yet some interesting questions are still missing. For instance, where is the research of AD and related dementias going in the next few years? What is accurate diagnosis and personalized therapy of basic pathophysiology of AD and related Dementias in the future? What are neurocognitive mechanisms of the neural mapping, which oversee the progress of dementia? What are possible precautionary measures of early dementia, and perceptions into effective therapy for early and advanced dementias? We hope that, in the future topic collections, we would like to see more articles that are imaginative and bring a novel perspective.

## Author contributions

G-PL: Writing—original draft. SW: Writing—review & editing.
